# Feasibility of ^177^Lu activity quantification using a small portable CZT-based gamma-camera

**DOI:** 10.1186/s40658-023-00602-2

**Published:** 2024-01-03

**Authors:** Daniel Roth, Erik Larsson, Joanna Strand, Michael Ljungberg, Katarina Sjögreen Gleisner

**Affiliations:** 1 Medical Radiation Physics, Lund, Lund University, Lund, Sweden; 2 Department of Radiation Physics, Skåne University Hospital, Lund, Sweden; 3 Department of Clinical Sciences Lund, Oncology, Lund University, Lund, Sweden; 4 Department of Hematology, Oncology and Radiation Physics, Skåne University Hospital, Lund University, Lund, Sweden

**Keywords:** Activity quantification, Hand-held gamma-camera, ^177^Lu, Molecular imaging, CZT

## Abstract

**Background:**

In image processing for activity quantification, the end goal is to produce a metric that is independent of the measurement geometry. Photon attenuation needs to be accounted for and can be accomplished utilizing spectral information, avoiding the need of additional image acquisitions. The aim of this work is to investigate the feasibility of ^177^Lu activity quantification with a small CZT-based hand-held gamma-camera, using such an attenuation correction method.

**Methods:**

A previously presented dual photopeak method, based on the differential attenuation for two photon energies, is adapted for the three photopeaks at 55 keV, 113 keV, and 208 keV for ^177^Lu. The measurement model describes the count rates in each energy window as a function of source depth and activity, accounting for distance-dependent system sensitivity, attenuation, and build-up. Parameter values are estimated from characterizing measurements, and the source depth and activity are obtained by minimizing the difference between measured and modelled count rates. The method is applied and evaluated in phantom measurements, in a clinical setting for superficial lesions in two patients, and in a pre-clinical setting for one human tumour xenograft. Evaluation is made for a LEHR and an MEGP collimator.

**Results:**

For phantom measurements at clinically relevant depths, the average (and standard deviation) in activity errors are 17% ± 9.6% (LEHR) and 2.9% ± 3.6% (MEGP). For patient measurements, deviations from activity estimates from planar images from a full-sized gamma-camera are 0% ± 21% (LEHR) and 16% ± 18% (MEGP). For mouse measurements, average deviations of − 16% (LEHR) and − 6% (MEGP) are obtained when compared to a small-animal SPECT/CT system. The MEGP collimator appears to be better suited for activity quantification, yielding a smaller variability in activity estimates, whereas the LEHR results are more severely affected by septal penetration.

**Conclusions:**

Activity quantification for ^177^Lu using the hand-held camera is found to be feasible. The readily available nature of the hand-held camera may enable more frequent activity quantification in e.g., superficial structures in patients or in the pre-clinical setting.

## Introduction

The radionuclide ^177^Lu is increasingly used for radionuclide therapy owing to its favourable decay properties, with a half-life of 6.4 days, the emission of short-range beta-particles and gamma photons with energies suitable for gamma-camera imaging. ^177^Lu also has favourable labelling properties and is currently authorized for therapy of neuroendocrine tumours using [^177^Lu]Lu-DOTA-TATE and for castration-resistant prostate cancer using [^177^Lu]Lu-PSMA-617 [1–4]. A number of trials are ongoing, such as therapy of non-Hodgkin’s B-cell lymphoma using [^177^Lu]Lu-Lilotomab [5]. In addition, there are several pre-clinical studies involving ^177^Lu. Most of these treatments are administered intravenously, although local administrations of [^177^Lu]Lu-DOTA-TATE are also being investigated for treatment of neuroendocrine liver metastases [6]. Probe detectors for radio-guided surgery of neuroendocrine tumours have been used with [^111^In]In-DTPA-Octreotide as tracer [7], and more recent investigations include [^68^Ga]Ga-DOTA-TATE and [^90^Y]Y-DOTA-TOC [8, 9]. The application of portable intra-operative probes for ^177^Lu detection and activity quantification has been addressed earlier [10]. Our group has previously investigated a hand-held gamma-camera system and successfully characterized its performance in ^177^Lu measurement [11]. Compared to conventional probe detectors, the small gamma-camera has advantages of providing both spectrometric and spatial information. A portable imaging system may also give possibilities for pharmacokinetic studies, where its ease of access can enable more frequent measurements than regular camera systems, for pre-clinical animal studies, or for superficially located structures in patients.

The detector signal from probes or camera systems inevitably depends on the amount and composition of the tissue between the detector and the source region. Activity quantification aims at removing this dependence, to provide a signal that is directly comparable between measurements for different source region positions in tissue. Conversion from a measured count rate into activity requires several steps to be taken, such as corrections for photon attenuation, scatter, collimator penetration, and camera system sensitivity. For planar imaging, these corrections are not straightforward, and different methods have been proposed, including for example combined correction of attenuation and scatter based on the effective attenuation coefficient applied for an estimated source depth [12]. Explicit scatter correction can be applied using the triple-energy window (TEW) that relies on additional scatter-energy windows in the energy spectrum to estimate the scatter component [13], or by model-based methods that rely on pre-calculated scatter kernels [14]. For the images acquired with the hand-held camera, we aimed for a simple and fast method, avoiding complementary imaging for estimation of the source depth.

The dual photopeak area method proposed by Strand and Persson in 1977 can be used for estimation of the source depth for radionuclides with double photon emissions in their radioactive decay [15]. The method is based on the energy spectrum and uses the difference in photon attenuation of two energy peaks to estimate the source depth. Application for activity quantification from conventional NaI-based gamma-camera images has been made for ^123^I [15], ^67^Ga and different combinations of ^99m^Tc, ^67^Ga, and ^111^In [16]. As the decay of ^177^Lu includes two prominent gamma emissions with energies 113 keV and 208 keV, as well as characteristic X-rays at approximately 55 keV [11, 17], we hypothesized that the dual photopeak area method would be applicable for quantification of ^177^Lu, and that the method could then be extended to three energy peaks, thus forming a multi-photopeak method. For scatter correction, we focused on the TEW method, owing to its simplicity. However, as the hand-held camera system is based on a cadmium zinc telluride (CZT) crystal combined with pixelated anodes, its energy spectrum has characteristics that are different from NaI-based cameras. In particular, there are low-energy tails caused by an incomplete charge collection which varies with the photon interaction position within the crystal [18]. Application of the TEW method to ^177^Lu spectra thus poses special challenges due to the interference of counts from primary and scattered photons in the main and scatter-energy windows of the three energy peaks. An additional challenge for activity quantification is a non-negligible contribution from septal penetration and collimator scatter, which despite the use of parallel-hole collimators, makes the system sensitivity dependent on the source collimator distance [11].

The aim of this work is to investigate the feasibility of ^177^Lu activity quantification with the CZT-based hand-held camera system. An activity quantification method is developed based on the multi-photopeak method for estimation of the source depth and related attenuation correction, combined with the TEW method for scatter correction. Evaluation is made by phantom studies, by comparisons of in vivo activity quantification for patients by conventional and hand-held gamma-camera imaging, and by pre-clinical mouse-imaging with the hand-held system and a small-animal SPECT/CT system.

## Material and methods

### Image acquisitions

**Fig. 1:**
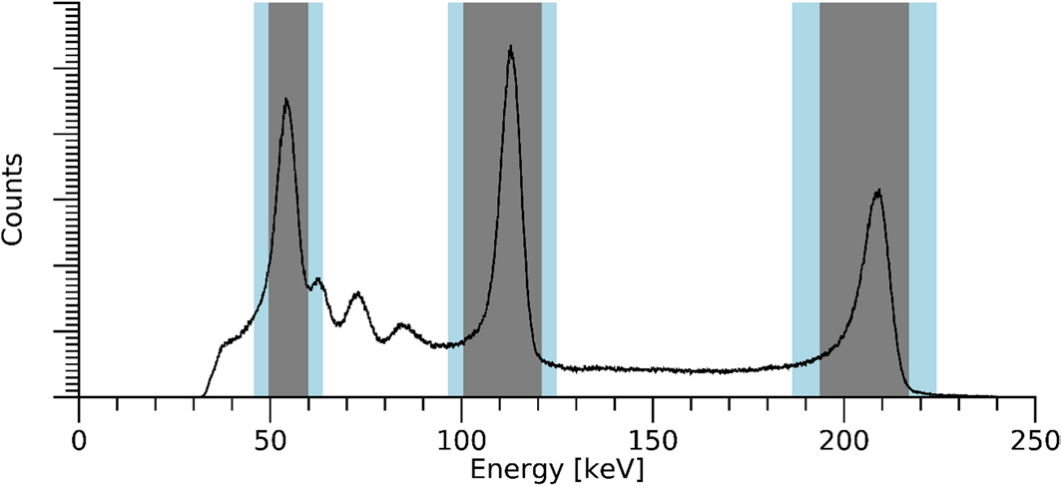
^177^Lu energy spectrum acquired with the hand-held camera. Photopeak energy windows are indicated in grey, and lower and upper scatter-energy windows in blue

The hand-held camera (CrystalCam, Crystal Photonics GmbH, Germany) is based on a single cadmium zinc telluride (CZT) detector module with a (39 × 39 × 5) mm^3^ crystal [11, 19]. An array of 16 × 16 anode contact pads is attached to the crystal with a pitch of 2.46 mm. Two collimators were used for ^177^Lu imaging, medium-energy general purpose (MEGP) and low-energy high-resolution (LEHR), with specifications given in “Appendix 1”. Data from all measurements were stored in a file format stating the number of recorded counts for each individual detector element, separated into in 0.1 keV wide energy bins from 0 to 250 keV. Using this format, images representing any energy window could be created from a single measurement. A correction for temperature-related drifts in energy calibration was first applied to the energy spectrum, and images were then extracted for photopeak energy windows set over the three most prominent peaks (Table 1, Fig. 1). Each of these images was then multiplied with a uniformity correction array, specific for the radionuclide, collimator, and energy window [11].

**Table 1:** Photon energies and energy windows used for ^177^Lu imaging. All values are given in keV

Name	Photon energy	Lower scatter window	Main window	Upper scatter window
55 keV	55.8	45.8–49.7	49.7–59.7	59.7–63.6
113 keV	112.9	96.5–100.5	100.5–120.8	120.8–124.8
208 keV	208.4	186.5–193.8	193.8–216.7	216.7–224.0

A resealable phantom was constructed in PMMA, consisting of a cylindrical cavity (20 mm inner diameter and 8 mm height) with PMMA walls (1 mm top and bottom thickness, 5 mm radial thickness) [11]. The phantom was filled with a solution of [^177^Lu]Lu-DOTA-TATE, and the inserted activity was measured using a traceable Secondary Standard Dose Calibrator (Southern Scientific, United Kingdom). The distance dependence of the camera’s system sensitivity in air was determined by placing the phantom in the centre of the camera field-of-view (FOV) and acquiring images at source collimator distances between 0 and 160 mm. To better represent smaller uptake volumes, the distance dependence was additionally determined with a small phantom, consisting of a plastic test tube with an inner radius of 5.9 mm and half-spherical bottom. The tube was filled to a depth of 8 mm with a [^177^Lu]Lu-DOTA-TOC solution, and measurements were made at distances between 0 and 100 mm. The impact of scattering medium was assessed by means of a glass micropipette tube placed at a fixed source collimator distance with varying amounts of PMMA slabs placed in-between. To enable conversion between different types of soft-tissue like materials, the amount of medium was parametrized in terms of the depth and linear attenuation coefficient product ($d\cdot \mu$). The range of PMMA thicknesses was 0 mm to 90 mm, giving ($d\cdot \mu$)-values of between 0 and 2.1 ($\rho \hspace{0.17em}$ = 1.18 g/cm^3^, $\mu /\rho \hspace{0.17em}$ = 0.20, 0.16 and 0.13 cm^2^/g for 55, 113 and 208 keV, respectively).

**Fig. 2:**
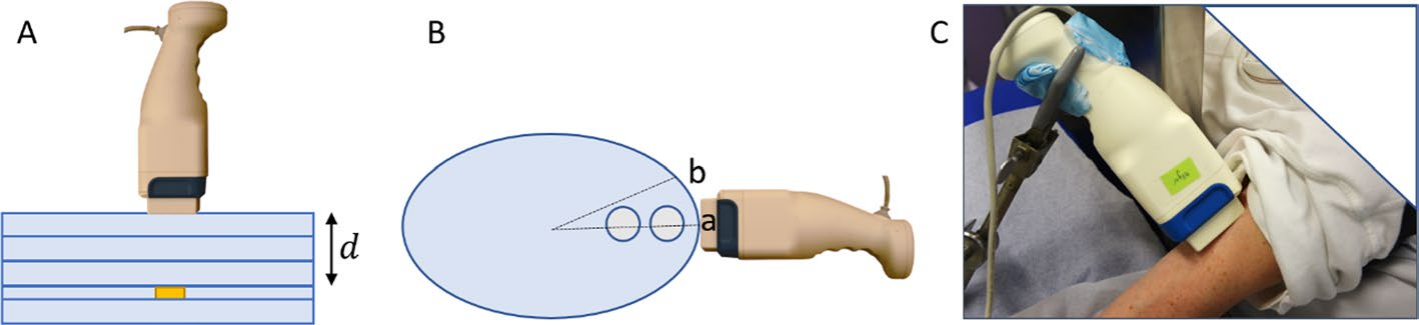
Measurement geometries used for evaluation: **A** cylindrical source in PMMA with incremental increase in the number of PMMA slabs. **B** water-filled phantom with spherical source placed at two different positions and with the camera placed in positions indicated (a) and (b). **C** Patient measurement

Evaluation was performed based on two sets of phantom measurements; one with the cylindrical source placed under PMMA slabs (Fig. 2A) and one with spherical sources in water (Fig. 2B). For the PMMA experiment, the cylindrical source was placed in a circular cut-out of a PMMA slab to properly model a geometry where a source is embedded in tissue. A further 25 mm of PMMA was placed below the source to yield realistic back scatter. The camera was centred over the source, PMMA was placed on top of the source (0 mm to 85 mm), and measurements made with the hand-held camera in contact with the PMMA. The spherical source experiment was made using two in-house 3D-printed spheres with volumes of 7.8 mL and 15.3 ml. An elliptical Jaszczak phantom filled with water was used as the attenuating medium. The elliptical phantom featured a sequence of points from its centre towards its edge along the long-axis where spheres could be mounted, and the two outermost positions were deemed reasonable for imaging with the hand-held camera. One sphere was placed in the phantom at a time during measurement. Measurements were made with the smallest sphere mounted in each of the two positions, while largest sphere could only be mounted and measured in the inner position. Measurements were made with the hand-held camera parallel to the phantom long-axis axis and 20° off-axis to vary the source depth further. The reference depths, defined as the distance between the phantom surface and the closest surface on the spherical inserts, were determined from a CT image of the phantom.

A comparison was made of the activity quantified from the hand-held camera and from conventional gamma-camera images for superficial lesions in two patients undergoing [^177^Lu]Lu-DOTA-TATE therapy [20]. In both patients, the lesions were located in the skeleton of the upper arm (humerus) as indicated in [^68^Ga]Ga-DOTA-TATE PET/CT images. Following the treatment protocol, planar anterior–posterior whole-body imaging was performed using MEGP collimators (Discovery 670, GE HealthCare, USA) at 1 h, 24 h, 96 h and 168 h after the 7.4 GBq [^177^Lu]Lu-DOTA-TATE infusion. A CT localizer image was also acquired for the purpose of attenuation and scatter correction. Descriptions of the treatment and imaging protocols are given in [20, 21]. For the conventional gamma-camera images, activity quantification was accomplished by a pixel-based implementation of the conjugate-view method, as described earlier [22]. Briefly, the gamma-camera images were co-registered to the CT localizer and converted to activity maps using pixelwise attenuation and scatter corrections. In connection with the regular imaging sessions, measurements were also made with the hand-held camera focused on the lesion. Prior to hand-held imaging, the lesion was located and centred in the FOV using short measurements as guidance. Acquisitions were made both with MEGP and LEHR collimators, with an acquisition time ranging from 1 to 5 min giving on average 20,000 counts (4000–80,000 counts) in the 113 keV energy window. Measurements with the MEGP collimator were omitted for the 168-h time point due to low count rates and the lower system sensitivity for this collimator [11]. As the exact location of the lesion with respect to the patient skin was not known at acquisition, different acquisition angles were investigated by placing the camera both from inside and outside of the patient’s arm, and from the anterior direction (Fig. 2C). A lead shield was placed between the patient’s torso and arm.

Evaluation was also done in a pre-clinical setting. One BALB/c nude mouse (Janvier labs, France) was implanted subcutaneously with SaOS2 cells (ATCC, USA) on the right hind leg 3–4 weeks before the experiment. Approximately 20 MBq of a ^177^Lu-labelled antibody targeting a specific epitope on SaOS_2_ tumours was administered through tail-vein injection. The animal was sacrificed 72-h post-injection and then imaged with the hand-held camera and a pre-clinical SPECT/CT system (BioScan NanoSPECT/CT Plus, Mediso, Hungary). With the hand-held camera, two images were acquired with the LEHR collimator and four with the MEGP. Imaging time was on average 9.3 min (range 3.7–11.5 min), giving 62,000 counts on average (range 17,000–99000) in the 113-keV window. Whole-body SPECT (acquisition time of 22 min) and CT scans were acquired and reconstructed using the standard protocol.

The CZT-based hand-held gamma-camera has been referred to herein as ‘hand-held’ to concisely distinguish it from the other camera systems used. In contrast to this designation, most measurements with this camera were performed with it immobilized by a holder or stand (Figs. 2C and 9C).

### Activity quantification method

The underlying quantities in the multi-photopeak method were the set of region-of-interest (ROI) count rates ${R}_{i,{\text{meas}}}$, measured in each energy window $i$ over the respective photopeak (Fig. 1). The measured count rate for emission $i$ (55, 113, 208 keV) was compared to the respective modelled count rate ${R}_{i,{\text{model}}}$, which was formulated according to

1 $$\begin{array}{*{20}c} {R_{{i,{\text{model}}}} \left( {A,d} \right) = A \cdot \varepsilon_{i} \left( d \right) \cdot e^{{ - d \cdot \mu_{i} }} \cdot B_{i} \left( {d \cdot \mu_{i} } \right),} \\ \end{array}$$

where ${R}_{i,{\text{model}}}$ represented either the gross count rate, or the net count rate after scatter correction, depending on whether a pixelwise TEW method was applied. The parameter $A$ was the source activity, $d$ the source depth, ${\mu }_{i}$ the linear attenuation coefficient for the photon energy of the energy window, and ${\varepsilon }_{i}$ the distance-dependent system sensitivity. When gross-peak count rates were used, i.e. without application of TEW scatter correction, the factor ${B}_{i}$ corresponded to the build-up factor earlier proposed for scatter correction [12]. When TEW scatter correction was used, ${B}_{i}$ was retained to adjust for tendencies to over- or under-estimate the scatter contribution. Based on experimental observation, ${B}_{i}$ was approximated as

2 $$\begin{array}{*{20}c} {B_{i} \left( {d \cdot \mu_{i} } \right) = 1 + k_{i} \cdot d \cdot \mu_{i} ,} \\ \end{array}$$

where ${k}_{i}$ was an energy-dependent correction factor. The distance-dependent system sensitivity ${\varepsilon }_{i}$ was modelled according to NEMA NU 1-2012 [23]:

3 $$\begin{array}{*{20}c} {\varepsilon_{i} \left( d \right) = c_{0,i} + c_{1,i} \cdot e^{{ - d \cdot c_{2,i} }} .} \\ \end{array}$$

For a given set of measurements, the source activity and source depth were estimated through a weighted least squares fit between ${R}_{i,{\text{meas}}}$ and ${R}_{i,{\text{model}}}$ using a gradient-expansion algorithm, with weighting by the inverse of the measured count rates as variance estimates. The initial estimates of $A$ and $d$ were calculated from

4 $$\begin{array}{*{20}c} {R_{{i,{\text{meas}}}} = A \cdot \varepsilon_{i} \left( {d_{{{\text{fix}}}} } \right) \cdot e^{{ - d \cdot \mu_{i} }} ,} \\ \end{array}$$

i.e. by applying the system sensitivity at ${d}_{{\text{fix}}}=20$ mm for each energy window $i$, and solving for $A$ and $d$ by linear regression of the logarithm of ${R}_{i,{\text{meas}}}/{\varepsilon }_{i}\left({d}_{{\text{fix}}}\right)$ as function of ${\mu }_{i}$.

In both Eqs. 1 and 3, the depth $d$ represented the distance between the face of the collimator and the nearest side of the source, rather than e.g. the effective depth used by Strand and Persson [15]. If the source thickness differed between the measured object and the phantom used to measure the camera sensitivity, differences in intra-source attenuation between the two sources was corrected for (“Appendix 4”).

### Implementation of activity quantification

From the in-air phantom measurements, the parameters of the distant-dependent system sensitivity ${\varepsilon }_{i}$ (Eq. 3) were determined for the two collimators, the three photopeak energy windows, with and without application of TEW scatter correction, and with the large cylindrical source and the small tube source. Circular ROIs were generated automatically based on the 113keV-window image, with centre placed at the centre-of-mass of the image counts and radius calculated as the source radius added by half the spatial resolution full-width at half-maximum for the actual source collimator distance [11]. For each set of distance-dependent data, parameter values were determined by an ordinary nonlinear least squares fit. The initial estimate of ${c}_{0,i}$ was set to the lowest measured sensitivity, while initial values for ${c}_{1,i}$ and ${c}_{2,i}$ were calculated by linear regression of the distance-versus-sensitivity data subtracted by ${c}_{0,i}$.

The factors ${B}_{i}$ and ${k}_{i}$ (Eq. 2) were determined based on the fixed distance measurements with varying amount of PMMA. Denoting the PMMA thickness as $s$ and measured count rates as ${R}_{i}\left(s\right)$ (either gross count rates or net count rates after TEW scatter correction), ${B}_{i}$ was calculated as

5 $$\begin{array}{*{20}c} {B_{i} \left( {s \cdot \mu_{{i,{\text{PMMA}}}} } \right) = \frac{{R_{i} \left( s \right)}}{{R_{i} \left( 0 \right)}} \cdot e^{{s \cdot \mu_{{i,{\text{PMMA}}}} }} } \\ \end{array}$$

where ${\mu }_{i,{\text{PMMA}}}$ is the linear attenuation coefficient for PMMA at the photon energy in window $i$. The factor ${k}_{i}$ was determined by an ordinary least-square fit to ${B}_{i}$ versus $s\cdot {\mu }_{i,{\text{PMMA}}}$ data.

**Fig. 3:**
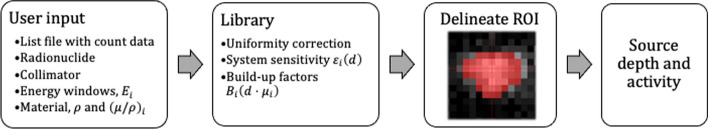
Flowchart of the workflow for estimation of activity and depth

The activity quantification method was implemented in IDL (Interactive Data Language, L3Harris Geospatial, USA) both as an automated procedure and a graphical user interface (GUI) (Fig. 3). Data of ${\varepsilon }_{i}\left(d\right)$ and ${B}_{i}\left(d\cdot {\mu }_{i}\right)$ for a specified radionuclide, collimator and set of energy windows were stored in a library. Additional specifications included the material (density and mass attenuation coefficient [24]) for determination of ${\mu }_{i}$ (Eqs. 1, 2 and 4) that could be set to water, soft-tissue or bone, or a mass-fraction based mixture of soft-tissue and bone. The GUI included capability to delineate ROIs, and the activity quantification could thus be made in one step.

### Evaluation

The accuracy in the estimated source depth and activity (Eq. 1) was investigated based on the measurements with varying source collimator distance and varying amount of PMMA. For the activity, the relative deviation from the activity determined in the dose calibrator ${A}_{{\text{ref}}}$ was determined according to

6 $$\begin{array}{*{20}c} {A_{{{\text{diff}}}} = \frac{{A_{{{\text{est}}}} - A_{{{\text{ref}}}} }}{{A_{{{\text{ref}}}} }},} \\ \end{array}$$

where ${A}_{{\text{est}}}$ is the estimated activity, decay-corrected to the times of dose calibrator measurements. The error in the estimated depth was quantified as the direct difference.

For the phantom measurements with spherical sources in water, ROIs were delineated manually to cover the source extension plus a distance of half the spatial-resolution FWHM at approximately the actual source camera distance. The relative deviation in the estimated activity (Eq. 6) and the absolute distance error were calculated.

For the patient measurements with the conventional gamma-camera, the tumour activity uptake was estimated by manual delineation of a ROI encompassing the tumour with margin to also include spill-out. Adjacent to this, a background ROI was delineated for estimation of under- and overlying activity and background subtraction. Operator sensitivity was addressed by letting two operators perform the ROI delineations. As the lesions were situated in skeleton, the mass attenuation coefficient used for activity quantification (Eq. 1) was calculated by assuming mass-fractions of 50% tissue and 50% cortical bone [25]. The activities derived from the conventional and hand-held camera images were compared using Eq. 6, with the conventional system held as reference ${A}_{{\text{ref}}}$. Each activity estimate for the hand-held camera was compared to the mean estimates from both operators for the conventional camera. Images for the 1-h time point were omitted from analyses, as the fast plasma turnover of [^177^Lu]Lu-DOTA-TATE early after administration could otherwise give different activity uptakes at the times of the two measurements [26, 27]. Moreover, as the count rate was modest at the last imaging time point, only the LEHR collimator was used owing to its higher geometric efficiency. The influence of the assumed attenuating material composition on the estimated activity and source depth was also investigated by examining these as functions of different tissue-bone fractions.

For the mouse measurements, the tumour activity uptake was estimated from the hand-held measurements with tissue as the assumed attenuating material. For the corresponding SPECT/CT image, the activity uptake was estimated by two operators, using manual delineations.

Sensitivities ${\varepsilon }_{i}$ (Eq. 3) determined from the large cylindrical phantom were used in near-all activity and depth estimations, with the exception of the mouse measurements where sensitivities obtained from the small tube were used instead, as this source was more similar in size to the implanted tumour.

## Results

**Fig. 4:**
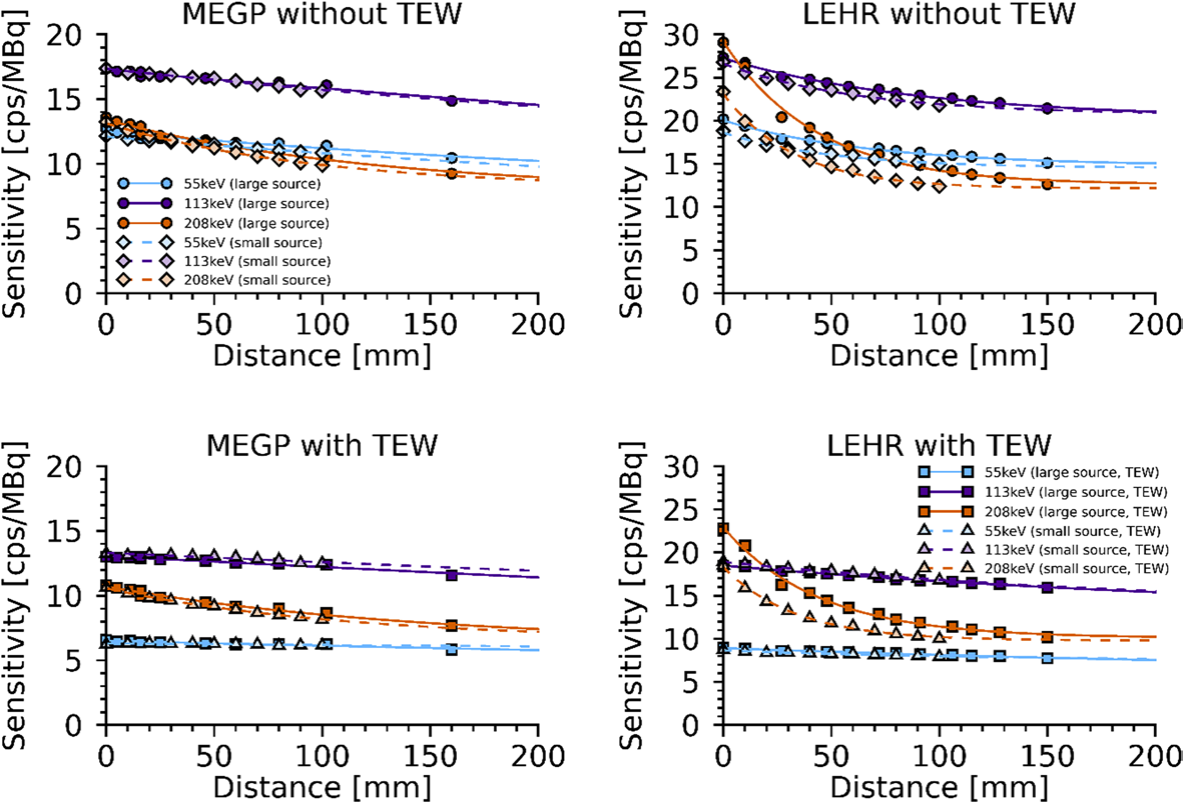
System sensitivity for different source collimator distances for LEHR and MEGP collimators, separated into the respective energy window. Upper panels are gross-peak count rates and lower panels are net count rates after TEW scatter correction

Figure 4 shows results of the in-air measurements of the system sensitivity for LEHR and MEGP collimators and the three energy windows, both with and without the application of the TEW method for scatter correction. As noted, the system sensitivity exhibited a clear distance dependence that was more pronounced for the LEHR collimator owing to its thinner septa and the larger amount of septal penetration, especially for 208-keV photons [11, 28]. Application of the TEW method reduced the peak count rate, and to some extent mitigated the distance dependence for the 55- and 113-keV windows. “Appendix 2” provides the obtained parameter values of Eq. 3, for both collimators and all energy windows. The sensitivities for the large cylindrical source and the small tube source agreed well, except for the sensitivity acquired for the 208-keV window with the LEHR collimator, where the smaller source and ROI radii decreased the proportion of detected septum-penetrating photons.

**Fig. 5:**
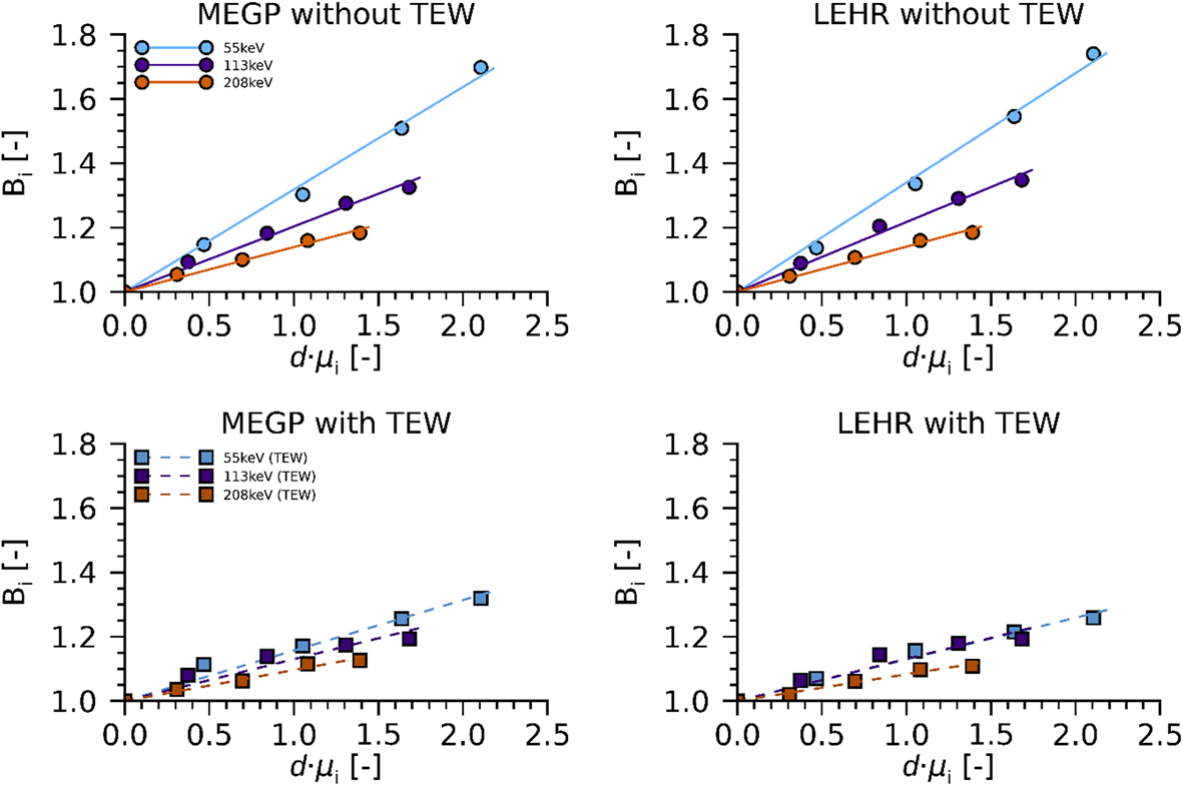
Factor ${B}_{i}\left(d\cdot {\mu }_{i}\right)$ (unitless) for the MEGP (left) and LEHR (right) collimators, for the three energy windows, as function of the depth and attenuation coefficient product ($d\cdot {\mu }_{i}$). Top row shows results using gross-peak count rates and bottom row results based on net count rates after TEW scatter correction

Figure 5 shows results from the PMMA phantom measurements and the estimate of the factor ${B}_{i}$ (Eqs. 2 and 5) when imaged at a fixed distance. Results are shown both with and without the application of the TEW scatter correction method. As noted, the amount of scatter was similar for the two collimators and decreased when the TEW method was applied. However, application of the TEW method did not fully compensate for the increased amount of scatter as the depth increased, and the factor ${B}_{i}$ was thus retained also when invoking the TEW method.

**Fig. 6:**
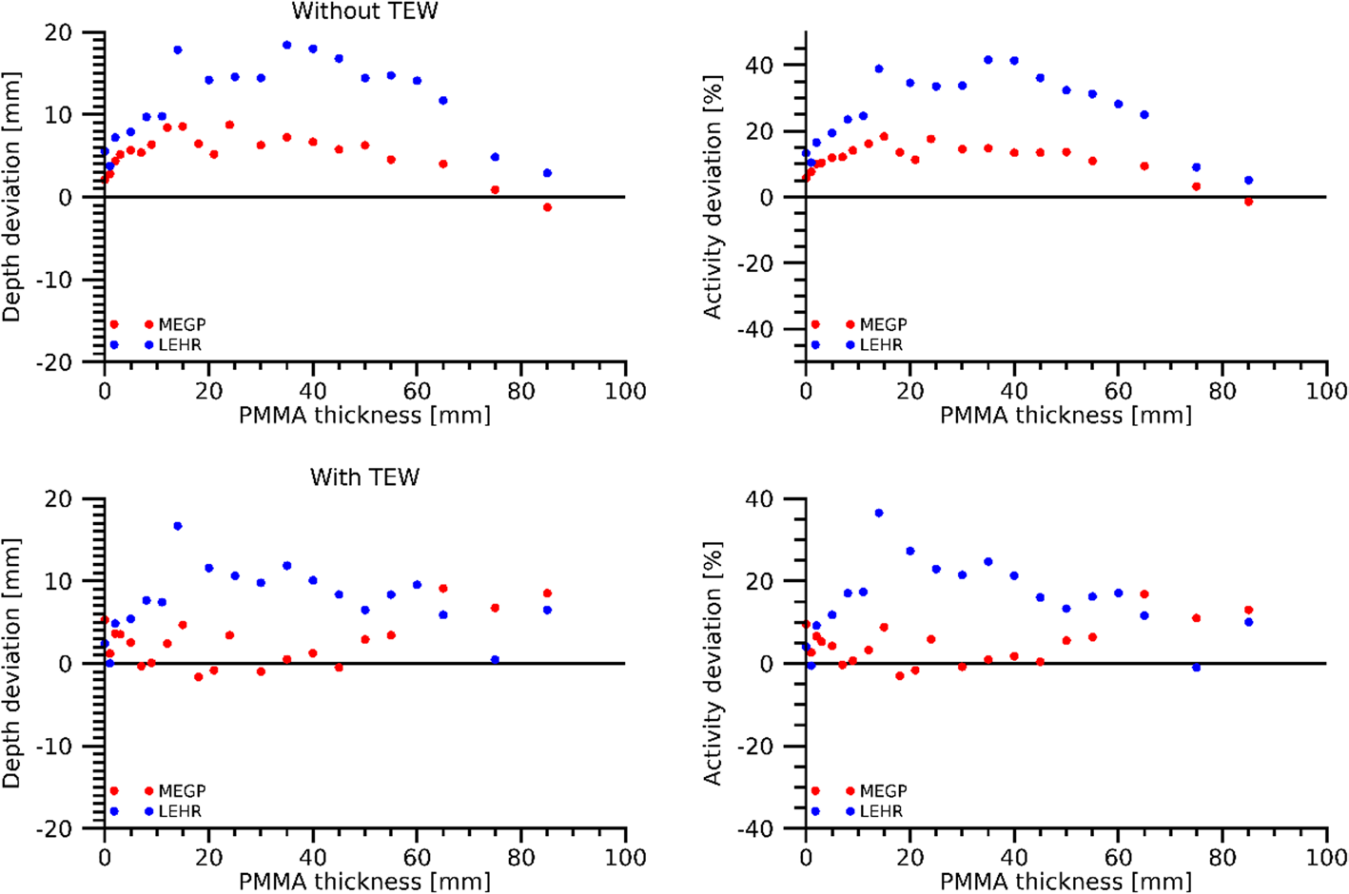
Deviation of the estimated depth (left panels) and relative deviation in the estimated activity (right panels) for the cylindrical source in PMMA. The deviations are shown as functions of the thickness of the PMMA between the source and collimator. Top panels are results based on gross-peak count rates, and bottom panels are results when TEW scatter correction is applied

The deviations of the estimated depth and activity for the cylindrical source measurements in PMMA are shown in Fig. 6. The mean and standard deviation of the relative activity deviations across all thicknesses were for the MEGP collimator 4.6% ± 5.1% when using TEW scatter correction and 11.4% ± 4.7% when using gross-peak count rates. For LEHR, the corresponding results were 16% ± 9.3% (TEW) and 26% ± 11% (gross-peak). For thicknesses within 50 mm, considered more realistic for practical use, the relative deviations when including TEW correction were 2.9% ± 3.6% and 17% ± 9.6% for the MEGP and LEHR collimators, respectively. Figure 6 demonstrates the strong positive correlation between the deviations of the estimated depth and activity. Including TEW correction, the estimated distance was for the MEGP collimator at maximum 9 mm from the true distance when calculated across all PMMA thicknesses, and 5 mm for thicknesses within 50 mm. For the LEHR collimator distance deviations of up to 17 mm were obtained, irrespective of PMMA thickness.

Table 2 shows results of the measurements of spherical sources in water. The source positioned at the smallest depth, which is most representative for practical use, gave activity deviations of − 4% and 7% with TEW correction for the MEGP and LEHR collimators, respectively, with corresponding depth errors of 2 mm and 8 mm. The mean and standard deviation in estimated activities across all five measurements when using the TEW correction were − 12.2% ± 5.0% (MEGP) and − 4.6% ± 9.5% (LEHR). When using gross-peak count rates, the corresponding values were − 9.6% ± 7.9% (MEGP) and − 2.1% ± 9.1% (LEHR).

**Table 2:** Results from the spherical sources in water. Camera positions (a) and (b) are indicated in Fig. 2. The reference depth denotes the distance from the phantom surface to the source surface

Collimator	MEGP					LEHR				
Sphere volume/mL	7.8			15.3		7.8			15.3	
Camera position	a	a	b	a	b	a	a	b	a	b
Reference depth/mm	24	67	71	61	65	24	67	71	61	65
Activity deviation w TEW/%	− 4	− 13	− 15	− 13	− 17	7	− 10	− 14	− 10	5
Activity deviation wo TEW/%	3	− 10	− 13	− 14	− 17	25	− 4	− 8	0	9
Depth deviation w TEW/mm	2	5	4	11	7	8	6	3	8	12
Depth deviation wo TEW/mm	5	9	5	8	5	11	12	7	13	13

**Fig. 7:**
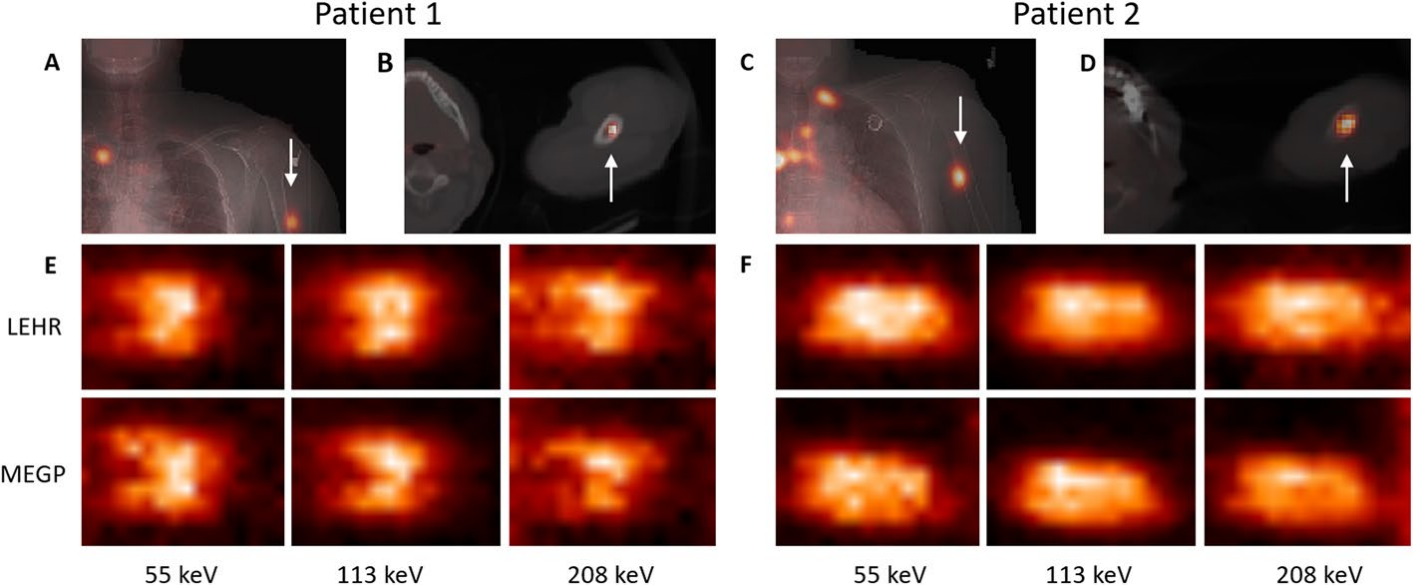
Example images for patient 1 (left) and patient 2 (right). Top row are **A**, **C** conventional gamma-camera images overlaid on the CT localizer, and **B**, **D** [^68^Ga]Ga-DOTA-TATE PET/CT images for tumour localization. Bottom panels show images from the hand-held camera acquired at day 1 with the LEHR and MEGP collimators, in the three energy windows. Images have been cropped for better visualization. Note that the orientation of the hand-held camera images depends on the camera positioning and does not correspond to that of the conventional camera systems. Panels** A** and** E** are adapted (cropped and re-arranged from material released under a Creative Commons Attribution 4.0 International License, https://creativecommons.org/licenses/by/4.0/) from [11]

For the patients, a total of eight hand-held measurements were made with the MEGP collimator and 13 with the LEHR collimator, distributed over the four imaging time points. Of these measurements, one acquisition was made with each collimator at 1 h (data not shown). Figure 7 shows example images of the two patients obtained using the conventional camera systems and the hand-held camera. In the hand-held camera images the activity distribution appeared considerably more heterogeneous than in the conventional gamma-camera images. Generally, the 113-keV energy window yielded the best visual image characteristics [11] and was thus used for ROI delineation. For the 208-keV energy window and the LEHR collimator a large component of septal penetration was detected, resulting in a higher background image signal. For both collimators, application of TEW scatter correction did not fully discriminate the scatter in the 55-keV energy window, resulting in a higher image background.

**Fig. 8:**
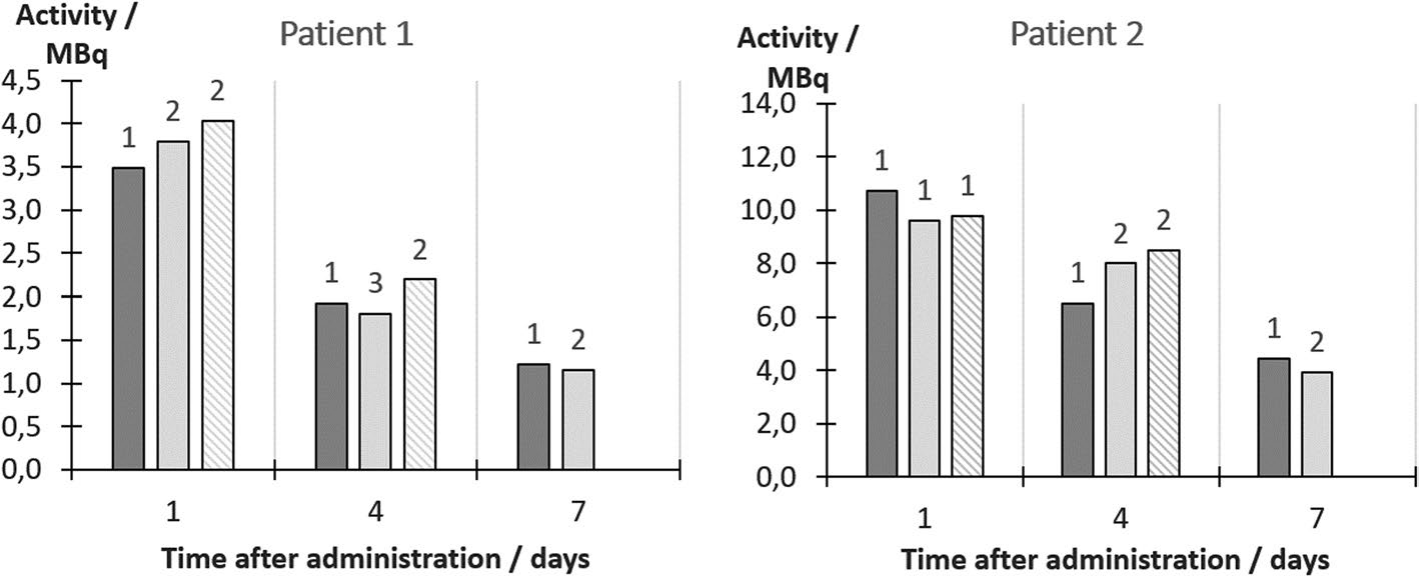
Activities estimated for superficial lesions in two patients, as function of the time after administration of [^177^Lu]Lu-DOTA-TATE. Dark solid bars are activities derived from conventional gamma-camera images, light grey and striped bars are results for the hand-held gamma-camera equipped with the LEHR (light grey) and MEGP (striped) collimators. When applicable, values for the hand-held camera represent the average from different acquisition angles, where the number of acquired angles is specified above the respective bar

For the conventional camera, the activities estimated from the two operators performing ROI delineation agreed to within 5.5%. Figure 8 shows the activities estimated from the conventional and the hand-held gamma-camera images, as function of time after administration for the two patients and using TEW scatter correction.

Overall, the estimated activities agreed well. Calculated across all imaging time points, the average deviation between the activities estimated with the conventional and hand-held camera with the MEGP collimator was 16% ± 18% (range − 9% to 49%). For the LEHR collimator, the average deviation was 0% ± 21% (range − 18% to 63%). The influence of the assumed fraction of tissue and bone for the attenuating material is shown in “Appendix 3”. The activity quantification was not overly sensitive to tissue fractions below approximately 60%, and the assumption of 50% tissue-bone composition was thus not critical.

**Fig. 9:**
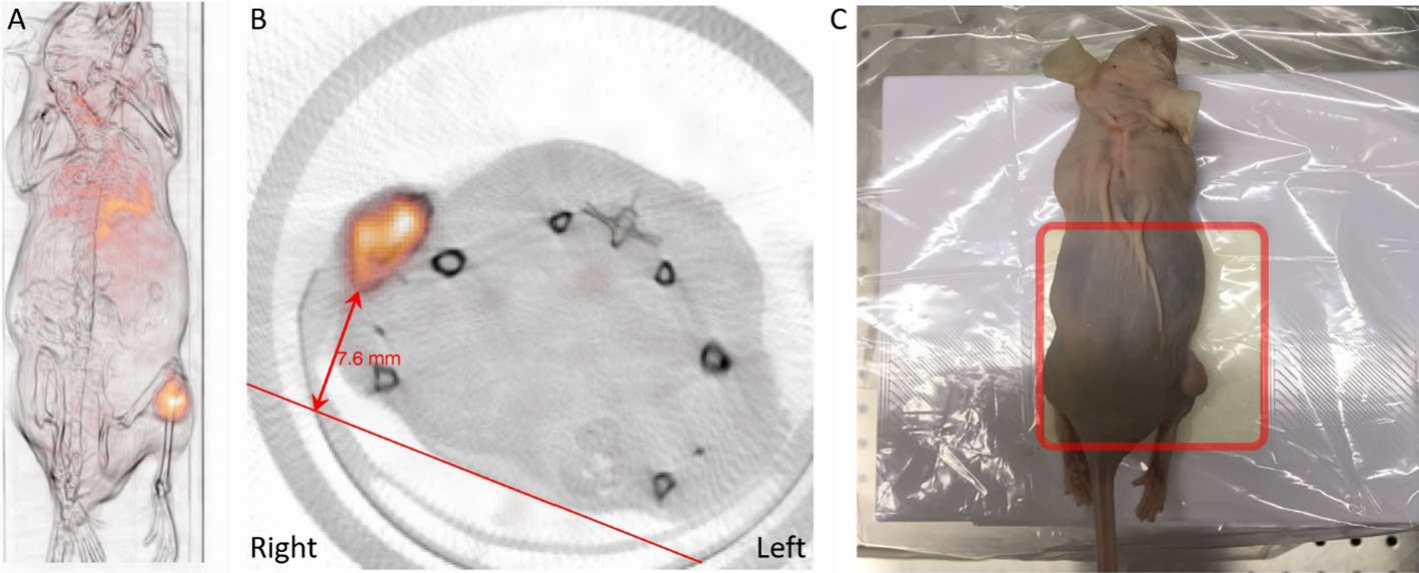
Illustration of the pre-clinical investigation. Panel A shows a SPECT/CT total-intensity projection, where the CT image has been high-pass filtered for visibility. Panel B shows an axial slice of the same data and the approximate collimator source distance for the hand-held measurement. Panel C shows a photograph of a hand-held camera measurement, with the animal placed on top of the collimator, as indicated by the red square

For the pre-clinical measurements, a total of six images were acquired with the hand-held camera. Figure 9 illustrates the imaging geometry for the hand-held camera measurement and the corresponding SPECT/CT image. The latter indicated a tumour depth of approximately 8 mm.

**Fig. 10:**
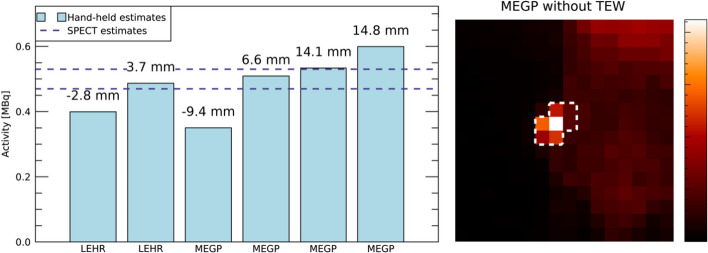
Estimates of tumour activity from the pre-clinical measurements (left) with data from the hand-held camera (bars) and estimates from the SPECT/CT by two independent operators (horizontal dashed lines). The depths estimated from the hand-held measurements are indicated above each corresponding bar. The right panel shows an image acquired with the hand-held camera, with the 113 keV window and MEGP collimator. The ROI used for activity quantification is indicated by a white dashed line

Figure 10 shows the activity uptake in the tumour estimated from the hand-held camera images and the SPECT/CT image. As the uptake region was relatively small, the sensitivities obtained from the small tube were used for the multi-photopeak method (Fig. 4). Overall, the hand-held activity estimates were similar to those obtained from SPECT/CT measurements. The mean deviation from the SPECT operator average was − 16% for the LEHR collimator (range − 25% to − 8%), and − 6% for the MEGP collimator (range − 34% to 13%). The estimated depths deviated somewhat from distances estimated from the SPECT/CT image, but these deviations had a limited impact on the activity estimates.

## Discussion

We find that ^177^Lu activity quantification for superficially located structures based on the planar images from the hand-held camera is feasible. The multi-peak method allows for attenuation correction without additional imaging for estimation of the tissue depth. Scatter correction can be accomplished with the TEW method, provided that an additional, experimentally determined correction factor is also applied. Owing to the characteristics of the energy spectra from CZT-based cameras, which include a tailing of counts with consequential crosstalk between the ^177^Lu photopeaks, the TEW method for scatter correction is not ideal. However, we find it applicable for the purpose of providing fast activity estimates, without the need of additional imaging for attenuation or scatter corrections. The possibility of storing measured data as files with energy spectra on a per-pixel level is found advantageous as it allows for detailed spectral analyses.

In our previous studies of ^177^Lu imaging with the hand-held camera, the LEHR collimator was found to give a higher sensitivity and better spatial resolution than the MEGP collimator, but with image-quality more severely affected by septal penetration by the 208-keV photons [11, 28]. The LEHR collimator together with the use of the 113-keV energy window was thus found best in terms of structure identification and general image characteristics [11]. In this work, focusing on image-based activity quantification, we find that the MEGP collimator provides more stable and accurate activity estimates than the LEHR collimator, especially in a well-controlled experimental setup (Fig. 6). The multi-peak method for estimation of the source depth essentially relies on the difference in attenuation coefficients between the three photon energies. The larger, and more varying activity errors obtained with the LEHR collimator are associated with the system sensitivity that exhibits a pronounced distance dependence for the 208-keV peak (Fig. 2), thus introducing a confounder when estimating the source depth. The underlying assumption of the multi-peak method, that 113-keV and 55-keV photons have a more pronounced depth dependence than 208-keV photons, thus becomes less well fulfilled. For conventional gamma-camera systems equipped with parallel-hole collimators, the system sensitivity has little or no distance dependence, and the multi-peak method may be of interest for ^177^Lu activity quantification of regions with limited superposition of activity in overlying tissues.

**Fig. 11:**
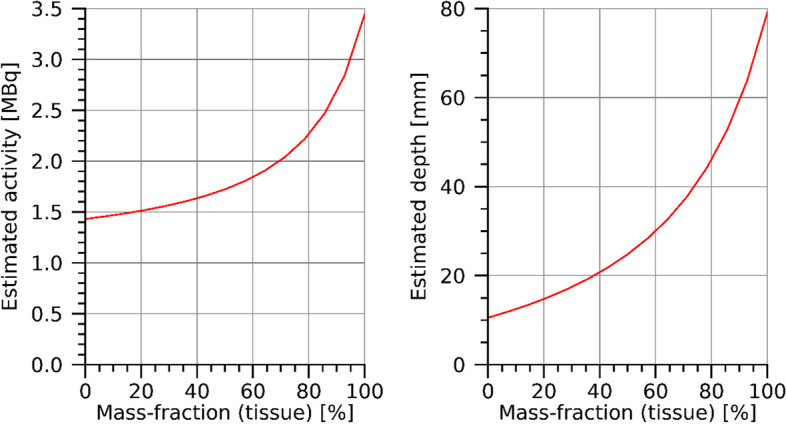
Estimated activity for one patient measurement with different assumptions regarding the fraction of soft tissue versus cortical bone (Eqs. 1, 2 and 4). For tissue fractions below 50%, the estimation of the activity is fairly insensitive to the assumed fraction. The depth estimation in contrast is more sensitive to the tissue composition, but is of limited interest in this activity quantification project

For patient measurements (Fig. 8) the hand-held camera provides activity estimates that are similar to those obtained from the conventional gamma-camera, with mean deviations within approximately 16%. As quantification based on the conventional camera images is considered more established it has been set as reference; however, it should be noted that these activity estimates also have uncertainties associated with them. Thus, the activities quantified with the two gamma-cameras are considered equivalent. The assumed mass-fraction of 50% tissue and 50% cortical bone is somewhat arbitrary, and changes to this mixture has a small effect on the activity estimates (Fig. 11, “Appendix 3”). Consequently, the average deviation in activity estimates (16% for MEGP, 0% for LEHR) should not be used as guidance when choosing an optimal collimator. For the hand-held system the differences between the two collimators are on average small, and in this case, the higher geometric efficiency of the LEHR collimator gives the advantage of allowing for faster measurement, especially at later times. However, as noted by the ranges of deviations, the MEGP collimator is considered to provide more robust activity estimates.

Pre-clinical measurements are an interesting application area for this method, where quick hand-held camera measurements could be used as complement to the longer SPECT/CT measurements. The results in Fig. 10 are encouraging for this application. The imaged tumour is smaller than the phantoms used in the sensitivity measurements, which may explain the variability in the activity estimates.

It should be noted that the two negative depth estimations in Fig. 10 are unrealistic, as this quantity is defined as the distance between the collimator face and the collimator-facing side of the source. Equation 1 can be evaluated for negative depths, although this represents an extrapolation outside the range of measured values. The algorithm used to fit modelled count rates to measured count rates did not support parameter constraints (e.g. that depth must be positive) and can therefore converge towards negative depths. Such behaviour could occur if the relative count rates between the three energy windows of a measurement deviate from what Eq. 1 predicts at positive depths (e.g. high count rates in the 55 keV window). This behaviour could be mitigated by using an algorithm with parameter constrains, or by characterizing and parameterizing factors that might influence the relative count rates (e.g. source and ROI size) and incorporate these into Eq. 1.

**Fig. 12:**
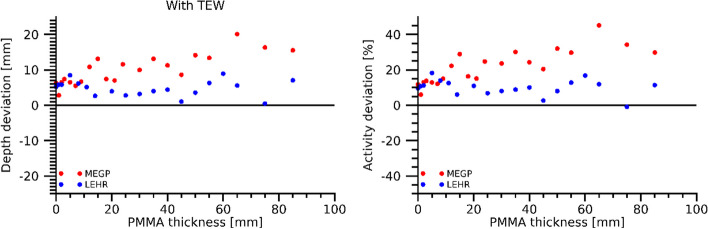
Error in estimated depth (left) and relative deviation in estimated activity (right) for the cylindrical source in PMMA, with the 208-keV window excluded. The TEW method has been applied, and deviations are shown as functions of the thickness of the PMMA between the source and collimator. The deviations across all thicknesses are obtained to 22% ± 9.6% (MEGP) and 10% ± 4.5% (LEHR)

The distance dependence of the system sensitivity (Fig. 2) was earlier investigated using Monte Carlo simulations [28] and was found to be mainly caused by septal penetration, photon scattering in the collimator and X-rays from the collimator. As the penetration fraction increases with photon energy, activity analyses of the data acquired as function of PMMA thickness have also been repeated when excluding the 208 keV window (Fig. 12, “Appendix 3”). For the LEHR collimator, the estimates of the activity and depth improve, while for the MEGP collimator, there is no benefit of excluding the 208 keV window.

As the counts from photons penetrating collimator septa have a broader spatial distribution than counts from geometrically collimated photons, the sensitivity is in practice dependent on the strategy for ROI delineation. Therefore, it is important to use a consistent ROI delineation approach when determining the sensitivity and the counts for an object to be quantified. However, the size of the source itself also influences the septum penetration proportion, as can be seen in Fig. 5 for the LEHR collimator and 208-keV window. This size dependency should resemble a typical recovery-curve, which flattens out for larger source sizes (see e.g. [29]). Consequently, for small sources, it is of greater importance to match the size of the source used for sensitivity measurements with the sizes expected in the intended application of the method. For the mouse measurements, the use of the small tube sensitivity source has thus been important, primarily for the LEHR measurements. For all other measurements, the exact size of the sensitivity source was found to be less important, either due to less septum penetration (MEGP measurements), or due to near-convergence in size dependency for the septum penetration fraction (measurements on large sources).

Interestingly, the ability to estimate the depth of a ^177^Lu source may have application also for radio-guided surgery of ^177^Lu-labelled compounds. The maximum distance error of 5 mm at a 5-cm source depth appears promising. Bugby et al. demonstrated the use of a portable gamma-camera equipped with a pinhole collimator to estimate the source depth based on stereoscopic imaging [30]. Acquisition of images from different angles could be applied also for the camera used herein, which could possibly give improved accuracy in position estimates from our multi-photopeak method. Stereoscopy for depth estimation may be preferable particularly if the tissue composition is uncertain, as depth appears to be more sensitive than activity towards tissue composition in the multi-photopeak method (Fig. 11, “Appendix 3”).

## Conclusions

Activity quantification of ^177^Lu based on the planar images from the hand-held camera is feasible. The multi-peak method allows for attenuation correction without additional imaging for estimation of the tissue depth. The portable system is easily accessible and can enable more frequent measurements than regular camera systems, for pre-clinical animal studies, or for superficially located structures in patients.

## Data Availability

The datasets used and analysed during the current study are available from the corresponding author on reasonable request, Daniel Roth (daniel.roth@med.lu.se).

## Appendix 1

See Table 3.

**Table 3:** Collimator specifications. All values are given in millimetre. Table adapted from [11]

Name	Hole length	Wall thickness	Hole width	Material	Hole shape
MEGP	11.5	0.96	1.50	Lead	Circular
LEHR	22.6	0.23	2.23	Lead	Square

## Appendix 2

Parameters obtained for the system sensitivity as function of source collimator distance (Eq. 3) (Table 4).

**Table 4:** Parameters describing the system sensitivity as function of source collimator distance

	Energy window [keV]	${c}_{0}$ [cps/MBq]	${c}_{1}$ [cps/MBq]	${c}_{2}$ [mm^−1^]
MEGP (gross)	55	6.8	5.7	0.0025
Large cylinder	113	− 1.2	18.4	0.0008
	208	7.8	5.6	0.0079
MEGP (net, TEW)	55	1.6	4.9	0.0008
Large cylinder	113	− 12.1	25.1	0.0003
	208	6.3	4.4	0.0067
LEHR (gross)	55	14.7	5.3	0.0143
Large cylinder	113	20.2	7.0	0.0107
	208	12.6	16.6	0.0232
LEHR (net, TEW)	55	5.5	3.4	0.0027
Large cylinder	113	12.4	6.1	0.0036
	208	10.1	12.8	0.0227
MEGP (gross)	55	5.5	6.6	0.0021
Small tube	113	9.4	8.0	0.0023
	208	8.1	5.1	0.0101
MEGP (net, TEW)	55	5.7	0.6	0.0026
Small tube	113	4.7	8.6	0.0009
	208	6.5	4.0	0.0090
LEHR (gross)	55	14.5	4.1	0.0198
Small tube	113	20.7	5.9	0.0157
	208	12.1	11.0	0.0306
LEHR (net, TEW)	55	7.4	1.2	0.0082
Small tube	113	13.2	5.7	0.0046
	208	9.8	8.5	0.0300

Parameter values are given for MEGP and LEHR collimators, for gross count rates and net count rates after TEW scatter correction, and for the large cylindrical source and the small tube source

## Appendix 3

See Figs. 11 and 12.

## Appendix 4

Intra-source attenuation (or self-attenuation) was modelled as follows. Assume that the source has a constant thickness $t$ along the axis that the gamma-camera is pointing along. The linear attenuation coefficient of the source for photon energy $i$ is ${\mu }_{i}$. Let $x$ be the depth within the source. The attenuation term in Eq. 1 applies only for material between the source and the camera, and radiation emitted towards the camera from depth $x$ within the source will thus be attenuated by an additional factor ${e}^{-x\cdot {\mu }_{i}}$. Assuming that the activity concentration is uniform within the source, the net intra-source attenuation ${F}_{i}\left(t\right)$ then becomes

7 $$\begin{array}{*{20}c} {F_{i} \left( t \right) = \frac{{1 - \exp \left( { - \mu_{i} \cdot t} \right)}}{{\mu_{i} \cdot t}}.} \\ \end{array}$$

$\underset{t\to {0}^{+}}{{\text{lim}}}{F}_{i}\left(t\right)=1$ corresponds to no reduction in measured count rate due to intra-source attenuation, and e.g. ${F}_{i}=0.95$ corresponds to a 5% reduction in measured count rate. Both the modelled count rates (Eq. 1) and the camera sensitivity (Eq. 3) are parameterized in terms of the distance between the collimator face and the closest side of the source. When the thickness of a measured source differed from the thickness of the phantom used to measure the camera sensitivity, the difference in intra-source attenuation was corrected for by multiplying the modelled count rates (Eq. 1) by the ratio ${F}_{i}\left({t}_{{\text{O}}}\right)/{F}_{i}\left({t}_{{\text{P}}}\right)$, where ${t}_{{\text{O}}}$ is the assumed thickness of the measured object and ${t}_{{\text{P}}}$ is the thickness of the sensitivity phantom. As the sources used herein were small (low intra-source attenuation) and similar in size to the sensitivity phantoms, these corrections were small.

## Abbreviations

CT: Computed tomography
CZT: Cadmium zinc telluride
FOV: Field of view
FWHM: Full width at half maximum
GUI: Graphical user interface
LEHR: Low energy high resolution
MEGP: Medium energy general purpose
PET: Positron emission tomography
PMMA: Poly(methyl methacrylate)
ROI: Region of interest
SPECT: Single-photon emission computed tomography
TEW: Triple-energy window
